# Can Myokines Serve as Supporters of Muscle–Brain Connectivity in Obesity and Type 2 Diabetes? Potential of Exercise and Nutrition Interventions

**DOI:** 10.3390/nu17223615

**Published:** 2025-11-19

**Authors:** Heaji Lee, Yunsook Lim

**Affiliations:** 1 Department of Food and Nutrition, Kyung Hee University, 26 Kyunghee-Daero, Dongdaemun-Gu, Seoul 02447, Republic of Korea; ji3743@hallym.ac.kr; 2 Department of Food Science and Nutrition, Hallym University, Chuncheon 24252, Republic of Korea

**Keywords:** myokines, sarcopenia, cognitive function, obesity, type 2 diabetes, nutrition, exercise

## Abstract

Background/Objectives: Skeletal muscle–derived myokines have emerged as pivotal mediators of the muscle–brain axis, linking peripheral metabolic regulation with central nervous system function. These molecules may influence skeletal muscle maintenance, neuroplasticity, neuroinflammation, and cognitive performance, and their dysregulation is increasingly associated with metabolic and cognitive impairment. In obesity (OB) and type 2 diabetes mellitus (T2DM), dysregulated myokine profiles characterized by reduced levels of irisin, brain-derived neurotrophic factor (BDNF), and cathepsin B (CTSB) have been reported and may contribute to the development of both sarcopenia and cognitive impairment. This review aims to summarize current evidence on myokine alterations in OB and T2DM and to evaluate how exercise- and nutrition-based interventions may modulate the muscle–brain axis to support metabolic and cognitive health. Methods: This narrative review synthesizes experimental, clinical, and translational studies examining (1) alterations in circulating myokines in OB and T2DM, (2) associations between myokines, skeletal muscle function, and neurocognitive outcomes, and (3) the modulatory effects of exercise and specific nutrients on myokine-mediated muscle–brain communication. Results: Available evidence indicates that OB and T2DM are frequently accompanied by reduced circulating levels of beneficial myokines such as irisin, BDNF, and CTSB, which may impair skeletal muscle integrity and contribute to cognitive decline. Restoring favorable myokine signaling through physical activity appears to enhance skeletal muscle maintenance, neuroplasticity, and metabolic homeostasis. Emerging data further suggest that selected nutrients can mimic or potentiate some exercise-induced myokine responses, thereby supporting both muscle and brain function. Collectively, these findings imply that combined exercise and nutrition strategies may exert synergistic or additive effects by reinforcing inter-organ communication along the muscle–brain axis. Conclusions: This review outlines current evidence on myokine alterations observed in OB and T2DM and discusses how exercise- and nutrition-based approaches may modulate the muscle–brain axis to mitigate metabolic dysfunction and preserve cognitive health. Targeting beneficial myokine pathways through tailored lifestyle interventions represents a promising avenue to support both skeletal muscle and neurocognitive function in individuals with metabolic disease.

## 1. Introduction

Obesity (OB) and type 2 diabetes mellitus (T2DM) are major global health burdens, characterized by chronic metabolic dysregulation, systemic inflammation, insulin resistance, and mitochondrial dysfunction [1,2,3]. These conditions often coexist and are interconnected through shared mechanistic pathways, constituting a progressive metabolic continuum [1,2,3]. In this context, skeletal muscle wasting and cognitive impairment are increasingly recognized as important comorbidities associated with chronic metabolic disease. Sarcopenia contributes to reduced metabolic capacity, impaired glucose utilization, and elevated systemic inflammation, while metabolic dysfunction and neuroinflammation have been implicated in accelerated cognitive decline. These clinical observations highlight the broad systemic consequences of metabolic disease across both peripheral and central tissues [4,5].

Despite the clinical significance of sarcopenia and cognitive decline, there are currently no approved cures for these conditions. Therapeutic approaches for cognitive impairment remain limited, largely due to challenges such as the impermeability of the blood–brain barrier (BBB) and the poor absorption and limited therapeutic effects of central nervous system (CNS)-targeted drugs. Similarly, no pharmacological agents for sarcopenia have been approved by Food and drug administration (FDA), highlighting the urgent need for alternative therapeutic strategies.

Emerging research suggests that structured exercise and targeted nutritional strategies can enhance the secretion of muscle-derived factors known as myokines, which play key roles in neurogenesis, synaptic plasticity, and inflammation, thereby supporting the functional relevance of the muscle–brain axis [6,7,8]. While myokines may play a contributory role, this bidirectional relationship also involves classical neuroendocrine factors such as hypothalamic appetite regulation, peripheral insulin resistance, and limbic reward processing, indicating that myokines represent only one facet of a broader regulatory network [9]. Importantly, heterogeneous findings across studies indicate that myokine regulation may vary depending on metabolic context, disease severity, and physiological status [10]. By modulating myokine profiles, lifestyle-based interventions may exert additive or even synergistic benefits on both metabolic and cognitive health.

This review highlights the emerging possibility that myokines act as key contributors to muscle–brain communication in metabolic diseases, particularly OB and T2DM. The regulation of muscle-derived signaling is influenced by complex neuroendocrine and metabolic interactions. Based on current evidence, this review summarizes heterogeneous and still-uncertain alterations in myokine expression in OB and T2DM, and discusses their potential mechanistic relevance to both muscle and brain function within this broader regulatory network.

In addition, we address exercise- and nutrition-based interventions that may modulate myokine secretion or signaling as part of integrated strategies to support metabolic and cognitive health [11,12,13,14,15,16,17].

## 2. Muscle and Brain Alterations in OB and T2DM

OB and T2DM represent a progressive metabolic continuum driven by chronic low-grade inflammation, insulin resistance, and mitochondrial dysfunction [18]. Systemic alterations contribute to pathological dysfunction in multiple organs, including skeletal muscle and the brain, two key regulators of whole-body energy homeostasis [19,20].

Skeletal muscle, the primary site of insulin-stimulated glucose uptake, is one of the earliest tissues affected by metabolic overload. As OB progresses to T2DM, impairments in insulin signaling, mitochondrial respiration, and protein turnover lead to muscle atrophy and reduced functional capacity [21]. These changes accelerate sarcopenia and further exacerbate metabolic deterioration.

In parallel, the brain becomes increasingly vulnerable to metabolic stress. Impairments in cerebral glucose metabolism, neuroinflammation, and disruption of BBB integrity have been reported in both OB and T2DM [22,23]. Moreover, neuroendocrine circuits governing appetite, sleep, and reward behavior show dysregulation, reflecting the substantial neurological and behavioral components of metabolic disease [24]. Structural and functional impairments in key regions such as the hippocampus and hypothalamus contribute to cognitive decline and altered energy regulation [22].

Sarcopenia and cognitive impairment, two common complications of OB and T2DM, frequently develop in parallel, suggesting interconnected pathological mechanisms beyond age-related decline [4,25]. These dual impairments result in reduced mobility, quality of life, and elevated risk of neurodegenerative disease.

## 3. Potential Role of Myokines in Muscle–Brain Connectivity in OB and T2DM

Epidemiological and meta-analytic evidence consistently showed that individuals with sarcopenia are at a significantly higher risk of cognitive dysfunction [26,27]. Recent research supports the concept of a bidirectional communication network between skeletal muscle and brain, often referred to as the muscle–brain axis [4,5]. The inter-organ axis is thought to be mediated in part by muscle-derived myokines that may influence neural plasticity, neuroinflammation, and cognitive performance. In particular, irisin, brain-derived neurotrophic factor (BDNF), and cathepsin B (CTSB) enhance neurogenesis, synaptic plasticity, and anti-inflammatory responses, while myostatin and interleukin (IL)-6 are involved in proteolytic and catabolic processes [4,5]. Notably, irisin and CTSB can cross the BBB and exert direct effects on brain physiology [28,29].

In addition to myokines that directly modulate neural tissue, several muscle-derived or muscle-regulated factors such as apelin, meteorin-like (METRNL), and myonectin have been proposed to indirectly influence brain function by improving systemic metabolic homeostasis, enhancing glucose oxidation, modulating vascular function, and regulating inflammation [30]. Although the direct contribution of these factors to neurocognitive outcomes remains less well established, they represent emerging candidates within the broader muscle–brain communication network and warrant further investigation.

In metabolic disease such as OB and T2DM, muscle–brain connectivity may be altered, resulting in decreased expression of neuroprotective myokines and elevated levels of pro-inflammatory mediators [31]. Molecular alterations associated with metabolic stress and myokine dysregulation may contribute to impaired muscle–brain communication. Importantly, these changes may interact with coexisting metabolic disturbances including insulin resistance, chronic inflammation, and vascular dysfunction to exacerbate functional deterioration in both systems [5,25,32]. Given the limited effectiveness of pharmacological treatments for either sarcopenia or cognitive impairment, the muscle–brain axis has emerged as a potential therapeutic target. Understanding how this axis is altered across disease progression provides a foundation for the development of integrated interventions to protect both muscle and brain health in metabolic disorders. To better clarify therapeutic opportunities, the following sections examine alterations in myokine expression in OB and T2DM, with particular emphasis on the potential role of interventions aimed at enhancing myokine secretion and signaling (Table 1). Such interventions may not only contribute to the prevention of disease onset but also offer therapeutic benefits by restoring muscle–brain communication and attenuating the pathophysiological consequences of metabolic dysregulation.

### 3.1. Biphasic Regulation of Myokines Across Disease Progression

A subset of myokines shows context-dependent or biphasic changes during the progression from OB to T2DM [9,10]. In early metabolic overload, transient increases in certain myokines may reflect adaptive responses that help preserve mitochondrial function, metabolic flexibility, and neuromuscular homeostasis. However, with prolonged insulin resistance and chronic inflammation, these compensatory responses become blunted or dysregulated, which may contribute to impaired muscle–brain communication and increased susceptibility to metabolic and cognitive decline [9,10].

Among the affected factors, BDNF, irisin, and FGF21 are noteworthy because they participate in both skeletal muscle metabolic regulation and neural processes relevant to cognition. Their ability to cross or influence the BBB suggests potential neuro-modulatory functions in metabolic disease. Although growing experimental and clinical evidence supports their role in the muscle–brain axis, direct links between their modulation and measurable cognitive outcomes in OB and T2DM remain under active investigation [56,57,58].

Given their dual metabolic–neurological actions and dynamic regulation across disease progression, these myokines represent promising candidates for biomarker development and therapeutic intervention. The following subsections describe their biological properties and disease-associated alterations in more detail.

#### 3.1.1. BDNF

BDNF, a member of the neurotrophin family, is indispensable for neuronal survival, synaptic plasticity, and cognitive function [33]. While its primary source is the CNS, skeletal muscle has emerged as an additional site of BDNF production, particularly under contractile or metabolic stimuli [33]. Muscle-derived BDNF promotes mitochondrial biogenesis, enhances fatty acid oxidation, and regulates myoblast differentiation through activation of AMPK signaling [34].

Beyond these metabolic functions, BDNF has been explored as a potential contributor to communication between muscle and brain [35]. Circulating BDNF may cross the BBB under certain physiological conditions and may influence hippocampal neurogenesis and plasticity [33]. Although the contribution of skeletal muscle to circulating BDNF remains uncertain, increased muscular expression of BDNF during exercise could potentially enhance this signaling pathway, supporting the concept of a muscle–brain connection.

In metabolic disorders, BDNF expression exhibits vulnerability to chronic stress and disease progression [36]. Reductions in muscle- and brain-derived BDNF have been consistently observed in both experimental models and clinical studies, with lower levels correlating with insulin resistance, cognitive decline, and sarcopenia [33,36,37,38]. Our previous findings demonstrated that diabetic mice displayed markedly reduced BDNF in skeletal muscle, plasma, and hippocampal tissue, indicating systemic impairment across the muscle–brain axis [59]. Chronic metabolic stress and neuroinflammation may also impair BDNF–TrkB signaling in the CNS, contributing to reduced responsiveness despite the presence of circulating BDNF [58]. Such alterations could attenuate downstream pathways such as CREB activation and thereby weaken neuroplastic adaptations [58,60].

From a translational perspective, these features make BDNF a compelling biomarker for monitoring metabolic–neurocognitive deterioration, as well as a therapeutic target. Interventions such as physical activity and nutritional strategies that support BDNF signaling are under investigation, but further mechanistic and longitudinal studies are required to establish their translational value in OB and T2DM.

#### 3.1.2. Irisin

Irisin, a cleaved fragment of fibronectin type III domain-containing protein 5 (FNDC5), has been identified as a key exercise-induced myokine with pleiotropic metabolic and neurocognitive functions [61]. In skeletal muscle, irisin facilitates mitochondrial biogenesis, promotes fatty acid oxidation, and drives the browning of white adipose tissue, thereby enhancing systemic energy expenditure and glucose homeostasis [39,40]. Through activation of PGC-1α-dependent pathways, irisin also regulates myogenic differentiation and preserves muscle metabolic flexibility [40].

Beyond these peripheral actions, irisin exerts significant effects on the CNS. Importantly, irisin crosses the BBB and induces hippocampal BDNF expression, leading to enhanced synaptic plasticity, neurogenesis, and memory performance [41,62]. This capacity to couple muscle activity with higher-order cognitive function positions irisin as a critical effector of the muscle–brain axis [8].

Clinical and experimental evidence indicates that circulating irisin levels are reduced under metabolic stress, including OB and T2DM, with declines correlating with insulin resistance, sarcopenia, and cognitive dysfunction [62,63]. Conversely, exercise and certain nutritional interventions restore irisin expression, linking its modulation to both metabolic resilience and neuroprotection [41,64,65]. However, conflicting clinical findings have also been reported [66]. For example, higher circulating irisin levels have been associated with early cognitive deficits in patients with poorly controlled T2DM, suggesting that elevated irisin may, in some cases, reflect compensatory responses rather than protective activity [66]. Such heterogeneity underscores the complexity of interpreting circulating irisin as a biomarker.

From a translational perspective, irisin represents both a biomarker for metabolic-neurocognitive deterioration and a therapeutic target, where lifestyle or dietary strategies aimed at sustaining irisin secretion may yield dual benefits in the prevention and management of OB and T2DM. Further longitudinal studies are required to clarify whether changes in circulating irisin directly translate into cognitive benefits in humans.

#### 3.1.3. FGF21

FGF21 is a stress-responsive hormone primarily secreted by the liver but also expressed in skeletal muscle, where it functions as a metabolic regulator and myokine [67,68]. Within muscle, FGF21 enhances glucose uptake, fatty acid oxidation, and mitochondrial biogenesis, while simultaneously mitigating oxidative stress through AMPK/SIRT1 activation [42]. Importantly, in human skeletal muscle, FGF21 has been shown to directly promote glucose uptake and improve insulin sensitivity, supporting its physiological relevance in metabolic homeostasis [43]. These effects contribute to the preservation of metabolic homeostasis under nutrient overload or stress conditions.

In addition to its peripheral roles, FGF21 penetrates the BBB and modulates neuronal plasticity by activating IGF-1/CREB signaling, which subsequently induces BDNF expression [44]. This crosstalk highlights FGF21 as a molecular integrator of muscle–brain communication, linking energy metabolism with neurocognitive function [4].

Under metabolic disorders, FGF21 expression is paradoxically elevated in circulation but often accompanied by FGF21 resistance, characterized by impaired signaling in target tissues [45,46]. This resistance state correlates with insulin resistance, hepatic steatosis, and cognitive impairment. Previous studies indicate that FGF21 expression and circulating levels exhibit stage-dependent changes during metabolic disease progression [69,70,71]. In early or compensatory stages of OB and insulin resistance, circulating FGF21 was upregulated as an adaptive response to metabolic stress or lipid overload [69,70]. However, in more advanced stages such as overt T2DM, this increase is often accompanied by impaired downstream signaling referred to as FGF21 resistance resulting in diminished metabolic and neuroprotective efficacy [71]. These findings suggest that the physiological impact of FGF21 may vary according to disease stage, reflecting a shift from compensatory adaptation to functional resistance.

Nonetheless, pharmacological FGF21 analogs and exercise-based interventions have been shown to restore its metabolic and neuroprotective functions [44,67,72,73]. Collectively, these findings underscore the dual potential of FGF21 as a biomarker of metabolic stress and a therapeutic target, offering opportunities to simultaneously improve systemic metabolism and cognitive health in OB and T2DM.

### 3.2. Persistent Dysregulation of Myokines in Metabolic Disease

Persistent and pathological dysregulation of specific myokines constitutes a critical mechanism driving metabolic deterioration and disease progression. Among them, CTSB, IL-6, and myostatin represent prototypical examples showing sustained or maladaptive alterations under chronic metabolic stress. While IL-6 and myostatin are persistently elevated, contributing to systemic inflammation, insulin resistance, and muscle wasting, CTSB typically decreases in metabolic disorders such as OB and T2DM, which may potentially impair lysosomal homeostasis and attenuate neuroprotective signaling along the muscle–brain axis. Collectively, these dysregulated patterns reflect a shift from physiological to pathological myokine signaling, promoting both metabolic and neurocognitive decline.

#### 3.2.1. Cathepsin-B (CTSB)

CTSB is a lysosomal cysteine protease traditionally recognized for intracellular protein turnover, but more recently identified as an exercise-inducible myokine with implications for both muscle homeostasis and brain function [74,75]. In skeletal muscle, CTSB contributes to mitochondrial quality control, autophagy, and inflammatory regulation, particularly under conditions of metabolic overload [76]. Its upregulation with exercise supports muscle remodeling and resilience against catabolic stress [76].

Strikingly, CTSB is capable of crossing the BBB, where it enhances hippocampal neurogenesis and synaptic plasticity, partly through the induction of BDNF-related signaling pathways [4]. Experimental studies have demonstrated that increased CTSB activity is associated with improved memory and learning performance, linking its exercise-induced secretion to neurocognitive benefits [47].

In metabolic disease, alterations in CTSB expression or activity have been reported in muscle and brain, although the direction and magnitude of these changes remain inconsistent across studies. Such dysregulation has been linked with impaired muscle function, insulin resistance, and cognitive vulnerability [74,75]. Interventions such as aerobic exercise and dietary modulation have been shown to increase CTSB levels, and these responses may contribute to the maintenance of both muscle function and cognitive health in metabolic disease. Nevertheless, additional mechanistic and longitudinal human studies are warranted to determine whether CTSB could serve as a clinically informative biomarker or therapeutic target in OB and T2DM.

#### 3.2.2. IL-6

IL-6 was the first myokine to be identified and is known for its diverse biological activities, displaying both pro- and anti-inflammatory properties depending on the physiological context [77]. Under physiological conditions, it is predominantly secreted by contracting skeletal muscle during exercise, where it promotes glucose uptake via GLUT4, enhances fatty acid oxidation, and stimulates myogenic differentiation through PI3K/AMPK activation [48,49]. Importantly, muscle-derived IL-6 can cross the BBB, where it interacts with neurons and glial cells to facilitate synaptic plasticity, BDNF-related neurotrophic signaling, and cognitive enhancement [4]. These transient elevations are thus considered adaptive and beneficial responses to metabolic stress.

In contrast, during chronic OB and progression to T2DM, IL-6 expression becomes sustained and increasingly derived from adipose tissue, liver (Kupffer cells), and immune cells [50,61]. This persistent elevation shifts IL-6 toward a pro-inflammatory phenotype, driving low-grade systemic inflammation, insulin resistance, and skeletal muscle catabolism [77,78]. Mechanistically, chronic IL-6 suppresses insulin signaling by downregulating GLUT4 and IRS-1, while impairing neuronal excitability and synaptic integrity, thereby contributing to muscle wasting and neurodegenerative processes [50].

Taken together, IL-6 exemplifies a dual-function myokine: acutely elevated and muscle-derived IL-6 supports metabolic and neurocognitive health, whereas chronically elevated and systemically derived IL-6 fosters metabolic deterioration and neuroinflammation. This dichotomy highlights the importance of source- and context-dependent IL-6 signaling when considering therapeutic targeting strategies.

#### 3.2.3. Myostatin

Myostatin, a member of the TGF-β superfamily, is a well-established negative regulator of skeletal muscle growth and regeneration [54]. Under physiological conditions, basal levels of myostatin play a critical role in maintaining muscle homeostasis by constraining excessive hypertrophy and ensuring balanced turnover of muscle fibers [51]. This regulatory role is essential for preserving muscle quality and preventing aberrant energy expenditure.

However, in the context of chronic OB and T2DM, myostatin expression is markedly elevated in skeletal muscle and circulation, where it exerts deleterious effects on metabolic and neurocognitive health [52,53]. Elevated myostatin inhibits Akt/mTOR signaling, suppresses protein synthesis, and promotes ubiquitin–proteasome-mediated protein degradation, thereby accelerating muscle wasting and sarcopenia [51,54]. In addition, myostatin impairs insulin signaling by downregulating IRS-1 and GLUT4 expression, aggravating insulin resistance and glucose intolerance [55]. Beyond skeletal muscle, myostatin has been shown to affect the CNS by reducing neurogenesis and synaptic plasticity, contributing to cognitive decline observed in metabolic disease [79,80].

On the other hand, emerging evidence suggests that the role of myostatin may be more complex and context-dependent. For instance, higher circulating myostatin levels were associated with a lower amyloid burden in older adults, possibly through autophagy-mediated amyloid clearance [81]. Similarly, increased myostatin levels have been observed in physically active or non-frail individuals after exercise, suggesting that transient elevations may reflect adaptive muscle signaling rather than purely catabolic processes [82]. These findings indicate that myostatin may exert different effects depending on metabolic and neurodegenerative context, warranting further investigation into its dual roles in the muscle–brain axis.

Thus, basal myostatin appears to support homeostatic regulation, whereas chronically elevated myostatin in metabolic stress conditions may contribute to muscle atrophy and metabolic dysfunction [52,53,54,55,79,80,81,82]. Understanding this context-dependent regulation may help identify optimal strategies for targeting myostatin to preserve muscle mass and cognitive function in metabolic disease.

Taken together, currently available evidence suggests that myokines may exert context-dependent effects during metabolic disease progression. Some myokines show biphasic actions—promoting metabolic and neurocognitive benefits under physiological or transient stress, but leading to pathological outcomes when chronically elevated or secreted from extra-muscular tissues [59,62,67,74]. In contrast, other myokines act predominantly as pathological mediators, contributing to sustained inflammation, insulin resistance, and neurodegeneration irrespective of disease stage of OB and T2DM [50,52].

The dual role of myokines underscores those therapeutic strategies must be tailored to the biological properties of each factor, including its cellular origin, temporal pattern of secretion, and the stage of disease progression. Such precision is essential for harnessing their protective potential while minimizing the detrimental consequences of myokine dysregulation in OB and T2DM.

While the current review highlights myokines with proposed direct effects on the CNS, various other muscle-derived factors may indirectly influence brain function. Myokines including apelin, myonectin, meteorin-like, and SPARC may modulate systemic metabolism, appetite control, and sleep regulation, potentially contributing to muscle–brain cross-talk through metabolic and endocrine pathways [83]. However, their contribution to neural outcomes remains less clearly defined, and further research is needed to determine whether these myokines exert meaningful effects on the muscle–brain axis in metabolic diseases such as OB and T2DM.

## 4. Therapeutic Strategies to Enhance Myokine Signaling in the Muscle–Brain Axis During Metabolic Disease

### 4.1. Exercise

Exercise plays a crucial role in maintaining skeletal muscle function and enhancing cognitive health [84,85]. Both aerobic and resistance training stimulate the secretion of beneficial myokines, including BDNF, irisin, and CTSB, which facilitate communication between skeletal muscle and the brain. These myokines promote neurogenesis, synaptic plasticity, and cognitive resilience, thereby contributing to overall brain function [86].

Recent meta-analytic evidence supports the beneficial effects of exercise on cognitive function in older adults with mild cognitive impairment. These findings suggest that exercise type and intensity may differentially modulate the release of myokines that influence brain function through metabolic and neurotrophic pathways [87].

Aerobic exercise enhances energy metabolism and mitochondrial function through activation of AMPK/PGC-1α pathways [88]. This metabolic enhancement promotes the release of myokines such as irisin and BDNF, which have been shown to support hippocampal neurogenesis and cognitive function by enhancing synaptic plasticity [86].

Resistance training, which primarily targets muscular strength and endurance, also improves mitochondrial function and activates autophagy in skeletal muscle [89]. It increases IGF-1 secretion, which crosses the BBB and promotes neuronal survival, synaptic plasticity, and memory [90]. Resistance exercise also upregulates BDNF through the CREB and mTOR signaling pathways, supporting cognitive function and neuroprotection [91].

Both aerobic and resistance exercises share core benefits, including enhanced skeletal muscle function, improved insulin sensitivity, and reduced inflammation. These effects are particularly relevant for individuals with metabolic diseases such as OB and T2DM, where sarcopenia and muscle insulin resistance are prevalent [92]. Exercise also improves systemic metabolic homeostasis by enhancing glucose uptake, increasing mitochondrial efficiency, and reducing chronic low-grade inflammation [93,94]. In terms of cognitive health, regular physical activity promotes neuroplasticity and protects against cognitive decline through the upregulation of neurotrophic factors, notably BDNF and irisin [95].

In individuals with metabolic diseases, regular exercise either aerobic or resistance offers a dual approach to combating sarcopenia and cognitive decline [95]. The synergistic effects of different types of exercise, particularly when combined with nutritional interventions, offer a comprehensive therapeutic approach to improve muscle function and brain health [96]. By targeting distinct yet interconnected physiological pathways, exercise serves as a fundamental component in the management of OB, T2DM, and related complications.

Importantly, the type and intensity of exercise should be tailored according to both the stage of metabolic disease progression and the individual’s age [97]. In younger or middle-aged adults at early stages of OB or T2DM, structured aerobic and resistance training can effectively enhance muscle mass and strength, maintain favorable myokine profiles, and provide long-term protection against sarcopenia and cognitive decline [97].

In contrast, in older adults or patients with advanced diseases, the feasibility of high-intensity exercise may be limited due to frailty, comorbidities, or increased susceptibility to exercise-induced oxidative stress [98]. In such cases, lower-intensity physical activity combined with targeted nutritional interventions may be more appropriate, helping to preserve muscle function and neurocognitive health while minimizing physiological stress.

### 4.2. Dietary Intervention

Nutritional interventions may function either as primary therapeutic strategies or as complementary approaches to exercise.

The following subsections summarize specific dietary components proposed to enhance myokine secretion or signaling, which could in turn contribute to improved muscle and brain function.

#### 4.2.1. Vitamin A

Vitamin A, particularly in its bioactive form all-trans retinoic acid (ATRA), plays a regulatory role in cellular differentiation, lipid metabolism, and mitochondrial function [79]. In the context of OB and metabolic dysregulation, ATRA has been shown to modulate adipogenesis and improve systemic energy balance [99,100]. Notably, ATRA enhances the expression of irisin in myoblasts, suggesting a potential link to muscle-derived endocrine activity. However, further in vivo and clinical studies are required to determine its relevance to sarcopenia [100].

On the other hand, vitamin A also exhibits neuroprotective effects by supporting synaptic plasticity and attenuating neuroinflammatory processes [101]. Animal and human studies indicate that vitamin A supplementation may improve memory performance and prevent OB-related cognitive decline [101].

These findings collectively highlight its potential to alleviate metabolic complications through modulation of the muscle–brain axis.

#### 4.2.2. Vitamin D

Vitamin D deficiency is commonly observed in individuals with OB and T2DM, and has been associated with insulin resistance, systemic inflammation, and impaired muscle function [14,102]. Beyond its classical role in calcium homeostasis, vitamin D is now recognized as a key modulator of skeletal muscle health and inter-organ communication [103].

Experimental studies have shown that vitamin D supplementation attenuates muscle atrophy by downregulating proteolytic markers such as atrogin-1 and MuRF1, thereby preserving muscle mass and strength in metabolic disease models [15]. Importantly, vitamin D also modulates the expression of myokines, which are critical mediators of muscle–brain crosstalk.

In T2DM mouse models, vitamin D supplementation has been shown to increase the expression of irisin, a myokine known to promote mitochondrial function, fatty acid oxidation, and BDNF production in the brain [65]. Consistently, clinical trials have demonstrated that six months of vitamin D supplementation elevates circulating irisin levels in humans [104], suggesting a conserved role for vitamin D in enhancing myokine-mediated signaling.

In the CNS, vitamin D exerts neuroprotective effects by supporting glial cell function, maintaining synaptic integrity, and enhancing hippocampal signaling [105]. Vitamin D insufficiency has been linked to increased risk of cognitive impairment in both aging populations and individuals with metabolic disorders [16]. Moreover, higher concentrations of vitamin D in brain tissue have been correlated with better cognitive performance in older adults [106].

Collectively, current evidence suggests that vitamin D supplementation may help attenuate sarcopenia and cognitive decline associated with metabolic diseases. These effects are proposed to be partly mediated through modulation of myokine expression, although direct causal relationships remain to be established.

#### 4.2.3. Polyunsaturated Fatty Acids (PUFAs)

PUFAs, particularly omega-3 fatty acids, have been reported to exert anti-inflammatory effects and are associated with improvements in various metabolic parameters [107]. Numerous studies have demonstrated the beneficial impact of PUFA intake on metabolic disorders, including enhanced insulin sensitivity, reduced systemic inflammation, and improved lipid profiles [11,108].

In particular, PUFAs influence the expression of muscle-derived signaling molecules, such as irisin and FGF21 [64]. In a previous study, supplementation with 1250 mg of PUFAs three times daily significantly increased serum irisin levels in patients with T2DM [64]. Furthermore, dietary intake of omega-3 (*n*-3) PUFAs has been positively associated with serum BDNF concentrations in adolescents, suggesting a potential link between PUFA intake and neurotrophic support in the context of psychiatric and metabolic disorders.

These findings imply that PUFAs may influence the skeletal muscle–brain axis indirectly through modulation of key myokines and neurotrophic factors, which could have potential implications for the management of metabolic diseases.

#### 4.2.4. Protein and Peptides

Adequate protein intake is fundamental for maintaining skeletal muscle mass and function, particularly under metabolic stress conditions such as OB and T2DM [109]. Proteins rich in essential amino acids, particularly branched-chain amino acids (BCAAs), activate the mTOR signaling pathway and stimulate muscle protein synthesis [110]. In addition to supporting muscle anabolism, dietary proteins and their bioactive peptides have emerged as important modulators of the muscle–brain endocrine axis, with potential roles in regulating both metabolic and cognitive health [111].

Dietary protein not only enhances muscle protein synthesis but also facilitates the release of specific myokines such as irisin, BDNF, CTSB, and IL-6, which are proposed to mediate peripheral-to-central signaling along the muscle–brain axis [112,113]. These myokines influence key aspects of brain function, such as mood regulation, hippocampal neuroplasticity, and central energy homeostasis, all of which are frequently impaired in the context of metabolic disease [111,112,113].

Beyond total protein intake, specific amino acids have been explored as modulators of metabolic and neurocognitive health. BCAAs, particularly leucine, activate mTOR signaling to support muscle maintenance and may enhance exercise-induced secretion of beneficial myokines such as irisin and IL-6, linking them to improved metabolic capacity [110,114]. Notably, however, metabolic context appears to influence their physiological effects, as chronically elevated BCAAs are associated with impaired mitochondrial metabolism and insulin resistance in OB and T2DM [115].

Beyond BCAAs, additional amino acids with bioactive neurometabolic functions have gained attention. Taurine, a sulfur-containing amino acid, supports muscle integrity by reducing oxidative and inflammatory stress and modulating catabolic myokines such as myostatin [116,117]. These regulatory actions suggest a potential contribution to inter-organ signaling relevant to muscle–brain communication.

Creatine enhances phosphocreatine-dependent ATP regeneration, improves insulin sensitivity, and reduces myostatin signaling [118]. Notably, creatine has been shown to augment exercise-induced increases in BDNF and neurocognitive performance, indicating a potential role in enhancing muscle-derived neurotrophic pathways [119].

Similarly, β-alanine supplementation increases intramuscular carnosine availability, enhancing buffering capacity during exercise and supporting metabolic resilience [120]. Carnosine has also been implicated in neuronal protection and oxidative stress regulation, offering a complementary mechanism that may intersect with muscle-derived endocrine signaling [121].

Taken together, the amino acid composition of dietary protein could influence muscle metabolism and systemic adaptations in a composition-dependent manner, potentially acting through myokine-mediated pathways that affect metabolic and neural health. A more nuanced understanding of these nutrient-specific effects could support precision nutrition strategies tailored to individuals with OB and T2DM.

#### 4.2.5. Polyphenols

Polyphenols, including resveratrol and curcumin, are bioactive compounds found in various plant-based foods and have been extensively studied for their potent anti-inflammatory and antioxidant properties [122]. They activate cellular signaling pathways related to energy homeostasis such as AMPK/SIRT1 pathway, leading to the regulation of myokine secretion that mediates muscle–brain crosstalk [122,123,124].

Resveratrol, a polyphenol primarily found in red wine, grapes, and certain berries, has attracted considerable interest for its potential therapeutic effects in metabolic disorders due to its anti-inflammatory and antioxidative properties [125,126]. It has been shown to modulate the expression of myokines such as IL-6 and BDNF [125]. Specifically, resveratrol decreases IL-6 secretion, potentially enhancing muscle regeneration and improving insulin sensitivity in various in vitro and in vivo studies [127]. Resveratrol also crosses the BBB, where it can influence neuroplasticity and cognitive function [128]. By upregulating neurotrophic factors such as BDNF, resveratrol supports neuronal survival and offers neuroprotective effects, which may contribute to the prevention of neurodegenerative diseases, including Alzheimer’s and Parkinson’s diseases [129].

Curcumin is the active compound found in turmeric and has long been recognized for its potent anti-inflammatory, antioxidant, and neuroprotective properties [130,131]. It is particularly effective in modulating inflammatory responses and supporting the health of both skeletal muscle and the brain [132,133]. Curcumin has been shown to influence the release of myokines such as irisin, thereby contributing to improved muscle metabolism and regeneration [134]. Additionally, curcumin promotes the expression of neurotrophic factors, including BDNF, and attenuates neuroinflammation, which may enhance cognitive performance and provide protection against neurodegenerative conditions [135,136]. Its ability to modulate the gut–brain axis further reinforces its role in preserving brain function, particularly under metabolic stress [137].

Collectively, polyphenols such as resveratrol and curcumin exert antioxidant and anti-inflammatory effects that counteract the pathophysiological mechanisms underlying sarcopenia and cognitive impairment. In addition to their direct benefits on muscle metabolism and neurotrophic signaling, these compounds may potentially enhance the release of neuroprotective myokines, thereby strengthening muscle–brain crosstalk and amplifying their therapeutic potential for metabolic and cognitive health in the context of obesity and T2DM. In summary, both exercise and dietary interventions have been suggested as potential strategies to modulate myokine signaling within the muscle–brain axis in metabolic disease. While preliminary findings indicate possible benefits, the current evidence remains insufficient to demonstrate a direct causal relationship, particularly in humans. Therefore, further mechanistic and well-controlled clinical studies are warranted to determine whether such interventions can effectively preserve both metabolic and cognitive health. Aerobic and resistance training stimulate the release of neurotrophic and metabolically beneficial myokines, thereby counteracting sarcopenia and cognitive decline. Complementarily, specific nutritional interventions including vitamins, PUFAs, proteins, and polyphenols, further augment myokine secretion and support inter-organ communication. Given the bidirectional communication between skeletal muscle and the brain, lifestyle interventions including diet and exercise that attenuate diabetic muscle atrophy may have the potential to indirectly support neurocognitive resilience by possibly improving myokine signaling and reduced systemic inflammation. Importantly, the efficacy of these approaches is highly dependent on age and disease stage, underscoring the necessity of tailored interventions. When implemented in combination, exercise and nutrition offer synergistic benefits that reinforce muscle and brain health, highlighting their potential as integrated therapeutic strategies for mitigating the metabolic and neurocognitive complications of OB and T2DM.

Although several studies suggest that nutritional modulation can influence myokine secretion and related pathways, evidence directly linking these changes to improvements in muscle–brain communication remains limited. Further mechanistic and clinical studies are needed to clarify their translational relevance in metabolic disease.

## 5. Future Directions and Limitations

Despite the increasing prevalence of sarcopenia and cognitive dysfunction, definitive therapeutic options remain limited. Myokines have emerged as key mediators of muscle–brain crosstalk, suggesting a potential therapeutic relevance in OB and T2DM, although their functional contribution requires further clarification [138]. However, current evidence insufficiently describes context-dependent changes in myokine signaling throughout disease progression. Further characterization of these alterations may support development of targeted intervention.

Importantly, the optimal balance between exercise and dietary interventions may vary among individuals [139,140,141,142,143,144,145,146]. In middle-aged individuals or early-stage disease of OB and T2DM, resistance and aerobic exercise can effectively enhance muscle mass and strength, thereby improving myokine profiles and potentially preventing later-life functional decline [142]. Building and maintaining higher muscle mass and strength during these earlier stages may provide a physiological reserve that helps delay or prevent functional deterioration in older age or during advanced stages of disease progression [143]. While exercise confers substantial benefits to muscle and brain health, its implementation must account for the potential exacerbation of oxidative and metabolic stress, especially in susceptible populations including the elderly, patients with T2DM, or those with chronic low-grade inflammation [144,145]. In this context, concurrent nutritional interventions encompassing sufficient protein intake alongside targeted antioxidant and anti-inflammatory supplementation represent critical adjuncts that can enhance anabolic signaling, counteract exercise-induced cellular damage, and sustain long-term functional outcomes [142,143]. In patients with older adults or advanced disease stages, where intensive exercise may be challenging, dietary interventions often become the primary approach. Targeted nutritional support can preserve muscle mass, attenuate oxidative stress, and sustain cognitive function, partly through modulation of myokine secretion and signaling. Integrative strategies not only support the independent improvement of muscle and brain health but may also restore inter-organ communication mediated by myokines.

Myokine dynamics likely vary according to physiological and metabolic conditions [8,59]. The modulatory effects of exercise and nutritional interventions also differ according to the type, intensity, and timing of the applied strategy. Moreover, individual myokines display distinct regulatory patterns and exert differential functional contributions to skeletal muscle and brain physiology [10]. These findings collectively suggest that the muscle–brain axis is governed not by isolated factors, but through the integrated and potentially synergistic actions of multiple myokines. Elucidating these intricate mechanisms represents an important direction for future research aimed at clarifying the role of myokine networks in inter-organ communication.

Nevertheless, research on nutrition-driven modulation of the muscle–brain axis remains in its infancy, and mechanistic pathways are yet to be elucidated. These findings are largely supported by preclinical evidence, and direct causal proof in humans remains limited. Therefore, the specific contribution of myokines to cognitive improvement requires further confirmation in clinical studies. Furthermore, interindividual variability in disease onset and progression complicates the standardization of interventions. Stage-specific responsiveness of the muscle–brain axis must be taken into account when designing future therapeutic approaches. Longitudinal and mechanistic studies using integrative methods including omics technologies, animal models, and clinical trials are essential to clarify the regulatory dynamics of myokines and to establish effective, personalized interventions for preserving both metabolic and cognitive health.

## 6. Conclusions

**Figure 1:**
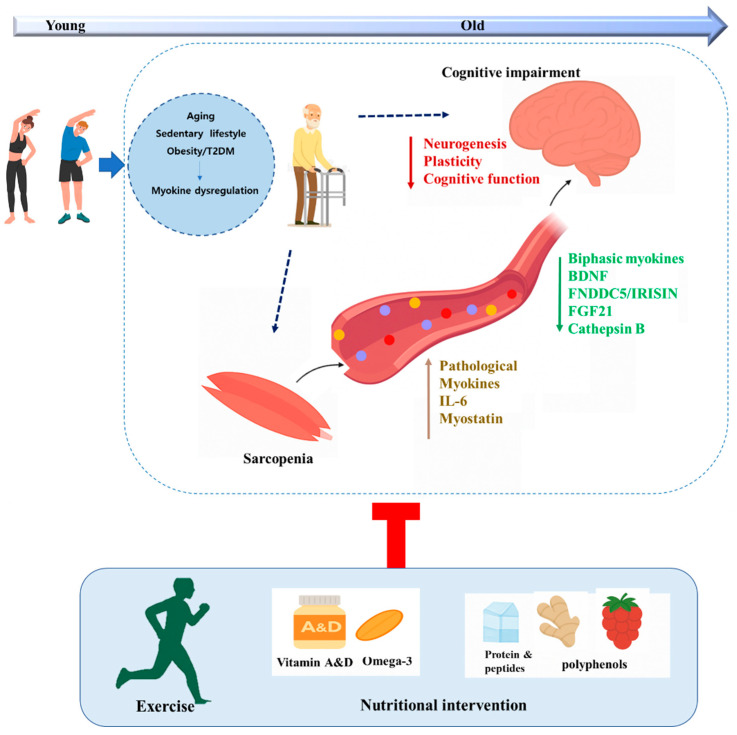
Potential therapeutic approaches to enhance myokine signaling for muscle–brain axis across OB and T2DM.

This review highlights the growing recognition of myokines as important mediators of muscle–brain communication in OB and T2DM. Altered myokine profiles may contribute to both metabolic dysfunction and neurocognitive decline, suggesting that restoring muscle-derived signaling could provide dual benefits. Although the precise mechanistic pathways remain incompletely defined, exercise and dietary interventions represent promising strategies to enhance myokine secretion and responsiveness (Figure 1). By supporting skeletal muscle function and neuroplasticity, these approaches may offer synergistic or additive effects through improved inter-organ connectivity. Continued clinical and mechanistic research is required to establish how myokine-associated interventions can be effectively translated into improved metabolic and cognitive outcomes in OB and T2DM.

## Figures and Tables

**Table 1:** Overview of muscle/liver-derived endocrine signals involved in the periphery-central crosstalk.

Circulating Factor	Origin (Expression Trend)	Target Organ	Effects on the Brain	Mechanisms of Action	Main References
Brain-derived neurotrophic factor	Skeletal muscle, brain (↑ → ↓)	Hippocampus, skeletal muscle	- Long-term potentiation and synaptic plasticity ↑ - Neuronal differentiation and cell survival ↑ - Hippocampal function ↑	- PI3K and ERK signaling pathway - PKB-CREB signaling	[33,34,35,36,37,38]
Irisin	Skeletal muscle, hippocampus (↑ → ↓)	Hippocampus, skeletal muscle	- Neuronal proliferation and differentiation ↑ - Synaptic function and memory ↑	- PKB and ERK signaling pathway - BDNF synthesis - PKA-cAMP-CREB signaling	[39,40,41]
Fibroblast growth factor 21 (FGF21)	Liver, skeletal muscle, adipose tissue (↑ → ↓)	Hypothalamus Adipose tissue, skeletal muscle	- Physical activity ↑ - Neurogenesis and neuronal survival ↑ - Insulin resistance↓ - Energy expenditure ↑	- PKB-CREB-mediated BDNF expression	[42,43,44,45,46]
CTSB	Skeletal muscle (↓)	Hippocampus, skeletal muscle	- Neurogenesis, memory, learning ↑ - Neuronal survival and anti-amyloidogenic activity ↑ - Clearance of α-synuclein ↑	- BDNF synthesis	[4,47]
IL-6	Skeletal muscle, adipose tissue, liver (Kupffer cells), macrophages (↑)	Hippocampus, skeletal muscle	- Neuroprotection and cognitive enhancement ↑ - Synaptic remodeling ↑ - Long-term potentiation ↑ - Maintenance of neuronal excitability ↑ - Cognitive dysfunction ↑	- JAK/STAT and AMPK pathway activation - Modulation of glutamatergic and GABAergic transmission - Interaction with BDNF - Neuroplasticity regulation	[48,49,50]
Myostatin	Skeletal muscle (↑)	Brain (cortex, hippocampus), skeletal muscle	- Cognitive impairment associated with sarcopenia ↑ - Memory deficits in AD model ↑ - Sarcopenia-induced neurodegeneration ↑	- Inhibition of myogenic genes (Myod, Myt5) via Akt/mTOR and FOXO1 pathway - Impaired muscle regeneration and satellite cell activation - Muscle wasting-mediated neurodegeneration	[51,52,53,54,55]

Arrows indicate expression trends of myokines in (OB) and T2DM. ‘↑ → ↓’ represents a biphasic response pattern characterized by an initial increase followed by a later decline under chronic metabolic stress, ‘↓’ denotes sustained reduction, and ‘↑’ sustained elevation.
